# Effects of Adverse Childhood Experiences on Depression Among Chinese College Students: A Mediated Moderation Model of “Shift-and-Persist” Strategy and Perceived Everyday Discrimination

**DOI:** 10.3390/bs15091167

**Published:** 2025-08-27

**Authors:** Yue Li, Xiaoyong Hu, Yuexin Ji, Hongjuan He

**Affiliations:** 1 Department of Psychology, Academy of Advanced Interdisciplinary Studies, Wuhan University, Wuhan 430072, China; 2 Education and Counseling Center for Psychological Health, Zhongnan University of Economics and Law, Wuhan 430073, China

**Keywords:** adverse childhood experiences, “shift-and-persist” strategy, perceived everyday discrimination, depression

## Abstract

(1) Background: the influence of adverse childhood experiences (ACEs) on depression is well-documented. Identifying protective factors to counteract ACEs’ detrimental effects is vital for public mental health. The efficacy of the “shift-and-persist” strategy as a mitigating factor against ACEs’ impact remains to be clarified. This study aimed to elucidate the relationship between ACEs and depression by testing a mediated moderation model, focusing on the moderating role of the “shift-and-persist” strategy and the mediating role of perceived everyday discrimination. (2) Methods: the research involved 1263 university students from Henan Province, China. A cross-sectional design was employed to test our theoretical model. Participants completed an online survey with assistance from trained research assistants. (3) Results: the study found that ACEs are a significant predictor of depressive symptoms in Chinese college students. The “shift-and-persist” strategy serves as a protective factor; high levels of this strategy weaken the predictive effect of ACEs on depression. Additionally, this strategy reduces the incidence of perceived everyday discrimination (PED) among those with ACEs, thereby alleviating depressive symptoms. (4) Conclusions: the “shift-and-persist” strategy effectively reduces PED and, consequently, depressive symptoms in individuals with ACEs, highlighting its potential as a protective factor in mental health interventions.

## 1. Introduction

Adverse childhood experiences (ACEs) are traumatic events occurring before age 18 (Felitti et al., 1998), including abuse (i.e., physical abuse, emotional abuse, and sexual abuse), neglect (i.e., physical neglect and emotional neglect), and family dysfunction (i.e., parental separation or divorce, household member being incarcerated, household mental illness, household substance abuse, and witnessing adult domestic violence). These events are common, with over 30% of U.S. individuals reporting at least one ACE (Davis et al., 2019; Jones et al., 2020) and more than 20% in China (Chang et al., 2019; Xiao et al., 2008; L. Zhang et al., 2020). In China, a meta-analysis shows 27% of children endure physical abuse, 20% emotional abuse, 9% sexual abuse, and 26% neglect (Fang et al., 2015). The impact of ACEs is significant, leading to various health issues like cardiovascular disease, hypertension, asthma, and mental health disorders (Carbone et al., 2019; Cheong et al., 2017; Fuller-Thomson et al., 2016; Godoy et al., 2021; Lietzén et al., 2021; Lipsky et al., 2022; Rogers et al., 2023; Su et al., 2015). These findings underscore the public health imperative to address ACEs and their long-term effects.

In response, researchers have developed and tested various strategies to mitigate the adverse consequences of ACEs. Among these, the “shift-and-persist” strategy has gained increasing attention for its demonstrated efficacy in buffering the effects of uncontrollable stressors, including ACEs (Choi et al., 2019; Crandall et al., 2019; Zheng et al., 2022). This strategy consists of two complementary components: shift—accepting and positively reappraising stressors that cannot be changed—and persist—maintaining optimism and finding meaning in life despite adversity. Empirical evidence shows that “shift-and-persist” can downregulate the hypothalamic–pituitary–adrenal (HPA) axis response, reduce perceived stress, and promote better long-term health outcomes in individuals with ACEs (E. Chen & Miller, 2012; Ertekin et al., 2021). Given these protective effects, investigating whether this strategy can attenuate the impact of ACEs on depression is crucial for identifying psychological resources that may reduce depression risk in this population.

### 1.1. Adverse Childhood Experiences and Depression

Depression, encompassing both symptoms and disorders, poses a significant global health challenge (Santomauro et al., 2021). The World Health Organization reports that 5.0% of adults worldwide, and 5.7% of those over 60, experience depression (WHO, 2023). In China, the prevalence of depression has risen from 3.22% to 3.99% between 1990 and 2017 (Ren et al., 2020).

A life course perspective suggests that early experiences shape health development and contribute to adult health disparities (R. M. Lerner et al., 2015; Masten & Barnes, 2018; Wadsworth, 1997). A foundational study revealed that individuals with four or more ACEs had a 4- to 12-fold increase in negative health outcomes, such as alcoholism, drug use, depression, and suicide attempts, compared to those without ACEs (Felitti et al., 1998). Further research indicates that ACEs significantly raise the likelihood of major depressive disorder, even when accounting for other factors (Cavanaugh & Nelson, 2022), and correlate with depressive symptoms in college students (Karatekin et al., 2018; Merians et al., 2019). Studies in the U.S. and China confirm ACEs’ enduring influence on adult depression (Desch et al., 2023; T. Zhang et al., 2023). Moreover, a meta-analysis shows that individuals who experienced any child maltreatment are 2.5 times more prone to depression, compared to those without ACEs (Gardner et al., 2019).

### 1.2. The Mediating Role of Perceived Everyday Discrimination

Adverse childhood experiences (ACEs) are closely linked to depression, primarily due to their association with chronic stress (Kong et al., 2021; Nurius et al., 2016). Stress is defined as a taxing relationship between an individual and their environment, often overwhelming their coping resources and leading to depression, particularly when experienced intensely during critical developmental stages (Lazarus, 1966; Vrshek-Schallhorn et al., 2020). During the developmental phase of active brain growth in childhood, ACEs can lead to heightened sensitivity in the hypothalamic–pituitary–adrenal (HPA) axis, amygdala, and hippocampus (Herzog & Schmahl, 2018). These potential influences contribute to heightened sensitivity, increased vigilance, and exaggerated responses to threatening stimuli in later stages of life (Teicher et al., 2016; Tottenham & Sheridan, 2009). Thus, those with higher ACEs tend to experience more chronic stress (Kong et al., 2021; Mc Elroy & Hevey, 2014; Vásquez et al., 2019). Additionally, perceived discrimination—a common stressor in those with ACEs—aggravates the situation. Research in North America demonstrates that childhood trauma is a significant predictor of perceived discrimination in adulthood, even when socio-economic factors are considered (Campbell et al., 2020; Matheson et al., 2019).

A growing body of research links perceived discrimination to an increase in depressive symptoms. Studies consistently show a significant correlation, with a U.S. survey among adolescents and a comprehensive European survey both reporting a strong positive relationship between discrimination and depression (Alvarez-Galvez & Rojas-Garcia, 2019; Dondanville et al., 2023; Hayes, 2017; Kim et al., 2019; Smith & Pössel, 2022). Meta-analyses support these findings, highlighting the adverse effects of discrimination on depression over a lifetime (Schmitt et al., 2014; Yip et al., 2019). In the Chinese context, Mao et al. (2022) found that perceived discrimination not only negatively affects mental health but also significantly and positively predicts suicidal ideation among economically disadvantaged college students.

Further investigations have explored how adverse childhood experiences (ACE) relate to perceived discrimination and depression. A study focusing on college students found that ACEs are significant predictors of psychological distress, including symptoms of depression, anxiety, and somatization, with discrimination serving as a mediating factor (Gangamma et al., 2020). Similarly, research from China suggests that ACEs can lead to depression through everyday stressors (Zheng et al., 2022). It implies that discrimination, as a prevalent stressor, may be a pathway by which ACEs adversely affect depression. Nonetheless, additional empirical studies are needed to confirm this hypothesis.

### 1.3. The Moderating Role of Shift and Persist

Numerous studies have established a link between ACEs and a heightened risk of depression in adulthood. Yet, it is noteworthy that not all individuals with ACE exposure develop mental health issues later in life; indeed, some demonstrate remarkable resilience and prosper despite adversity. This observation underscores the existence of protective factors that can counteract the negative impact of ACEs (Bonanno et al., 2011; Choi et al., 2019; Crandall et al., 2019; Mc Elroy & Hevey, 2014; Zheng et al., 2022). To fully understand the interplay of protective and risk factors during human growth and development, a comprehensive examination is necessary. Factors such as resilience, social support, and coping mechanisms interact dynamically, shaping an individual’s response to adversity (Sege & Harper Browne, 2017).

Identifying psychological resources that mitigate the detrimental effects of ACEs is crucial for promoting overall well-being and development. One such coping strategy is the “shift-and-persist” approach (E. Chen & Miller, 2012). It involves accepting and reappraising uncontrollable stressors (the “shift” component) while simultaneously maintaining optimism and seeking meaning (the “persist” component). For instance, when faced with failure, individuals employ cognitive reappraisal to accept the reality of their situation (the “shift”). Simultaneously, they extract valuable lessons from the experience, fostering optimism and purpose (the “persist”). By embracing this dual approach, individuals can effectively navigate stressors and cultivate resilience.

According to E. Chen and Miller’s (2012) conceptual model, the development of the “shift-and-persist” strategy primarily depends on the pivotal role of role models (e.g., parents, teachers, or other significant others). Role models foster this strategy through two mechanisms. First, by establishing secure attachment relationships, they cultivate children’s fundamental trust in others (Bowlby, 1988) and shape a positive worldview (e.g., optimistic beliefs), thereby laying the psychological foundation for the strategy (E. Chen & Miller, 2012). Secure attachment not only enhances children’s emotion regulation capacity (Cooke et al., 2019) but also strengthens their perceptions of environmental predictability and social support, which underpin adaptive adjustment in the “shift” dimension and meaning-making in the “persist” dimension. Second, role models transmit concrete skills through socialization: setting emotional expression rules (e.g., “anger can be expressed but not aggressively”) to guide socially acceptable emotion regulation (Gottman et al., 1996; Houltberg et al., 2016); responding constructively to children’s emotional distress (e.g., problem-solving assistance, encouragement of expression) to model adaptive coping strategies (Uyar et al., 2018); and demonstrating positive emotional expression in adversity to reinforce children’s learning of regulation strategies (L. Tan & Smith, 2019; Denham et al., 1997). Moreover, role models encourage a future-oriented perspective and a search for life meaning, thereby fostering optimism and meaning-seeking in the “persist” dimension (J. V. Lerner et al., 2021).

The “shift-and-persist” strategy has demonstrated positive health outcomes for indi-viduals facing uncontrollable stressors (Adesogan et al., 2023; E. Chen, 2012; L. Chen et al., 2019; Stein et al., 2022). Initially, this strategy was investigated to mitigate the adverse effects of poverty and economic stress on physical health (E. Chen, 2012; E. Chen & Miller, 2012; Kallem et al., 2013). Recently, scholars have expanded their investigation of the “shift-and-persist” strategy to explore its potential protective effects on the mental health of disadvantaged groups in various stressful contexts. Subsequent studies found that this strategy is beneficial for individuals who perceive discrimination. For instance, a survey revealed that the “shift-and-persist” strategy was associated with fewer depressive symptoms among Latino youth experiencing economic stress and discrimination (Christophe et al., 2019). Moreover, high levels of the “shift-and-persist” strategy were found to mitigate the negative impact of peer racial discrimination on depressive symptoms among Mexican–American youth (Christophe & Stein, 2022). Meta-analysis results also indicate that the “shift-and-persist” strategy is linked to fewer internalizing symptoms (Compas et al., 2017). According to E. Chen and Miller (2012), the “shift-and-persist” strategy reduces the perception of stress, ultimately diminishing acute physiological activation of the hypothalamic–pituitary–adrenal (HPA) axis and the sensorineural system (SNS). Consequently, it weakens the link between stressors and negative long-term health outcomes (E. Chen & Miller, 2012; Ertekin et al., 2021). Thus, the “shift-and-persist” strategy not only leads to adaptive down-regulation of the stress response system (E. Chen, 2012) but also serves as a protective factor buffering the effects of stress on health. Given the predictive role of stress response alterations in depressive symptoms (Slavich & Irwin, 2014; X. Tan et al., 2021), this strategy could be particularly valuable in lessening the impact of uncontrollable stressors like ACEs on depression.

Although direct empirical evidence on the protective mechanisms of the “shift-and-persist” strategy for the mental health of Chinese individuals with ACEs remains limited, indirect support can be drawn from related findings. Psychological resilience—conceptually similar to the “shift” component and defined as the capacity to recover and adapt quickly in the face of adversity—has been shown to offer substantial protection for this population. For example, resilience can buffer the detrimental effects of ACEs on attachment security, significantly reducing post-adversity increases in attachment anxiety and avoidance (J. Zhang et al., 2025). Another study revealed gender-specific pathways: in women, resilience mediated the link between abuse/neglect and mental health, whereas in men, it mediated the relationship between family challenges and mental health (Y. Chen et al., 2021). Collectively, these findings provide indirect evidence supporting the potential of the “shift-and-persist” strategy to protect the mental health of Chinese individuals with ACEs.

### 1.4. The Present Study

Extensive research has explored the association between ACEs and depression. However, significant gaps remain. First, few studies have investigated the impact of discrimination on depression among college students with ACEs. Early adulthood is a pivotal developmental stage where mental and physical health trajectories form (Essau et al., 2020; Hargrove et al., 2020). College students face various stressors, including perceived discrimination arising from social interactions (Y. Liu et al., 2020; Williams et al., 1997). Therefore, understanding the impact of perceived discrimination on the mental health of college students with ACE histories is crucial for their overall well-being. Second, not all individuals who experience ACEs develop depression. This highlights the need to identify psychological resources that can mitigate the adverse effects of ACEs (Carbone et al., 2019; Zheng et al., 2022). The “shift-and-persist” strategy, supported by both theory and empirical evidence, may offer such protection. However, empirical research in this area remains limited.

**Figure 1:**
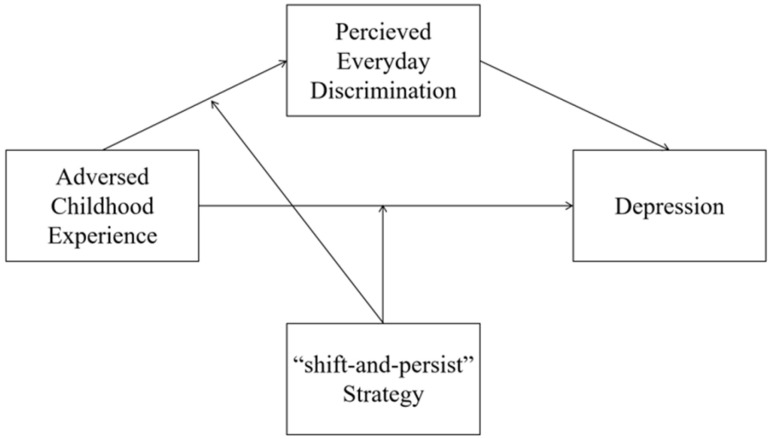
Conceptual model.

In summary, our literature review led to the development of a mediated moderation model (see Figure 1). Our study aims to determine whether the “shift-and-persist” strategy can reduce depression levels in Chinese college students with ACEs. Additionally, we examine whether “shift-and-persist” reduces perceived discrimination among individuals with ACEs, thereby weakening the link between ACEs and depression. We propose the following hypotheses:

**H1.**  *ACEs significantly predict depression, with individuals who have experienced ACEs exhibiting higher depression levels than those without such experiences.*

**H2.**  *The “shift-and-persist” strategy serves as a mediated moderator, reducing the association between ACEs and depression by lowering perceived discrimination levels among Chinese college students with ACEs, ultimately decreasing depression levels.*

## 2. Materials and Methods

### 2.1. Sampling and Participants

This study adopted convenience sampling and, in April 2024, distributed an online questionnaire link to students at Zhengzhou University, Henan Province, China. Recruitment information was disseminated in collaboration with relevant university departments and student organizations via their social media platforms and internal communication systems over a two-week period. The recruitment notice included a brief description of the study, a statement on privacy protection, and information on incentives for completing the questionnaire. All participants provided informed consent before beginning the survey. A total of 1320 students participated. During data cleaning, we excluded blank questionnaires, patterned responses, and cases with excessively short completion times, based on the following criteria: (1) Blank questionnaires: fewer than 50% of items completed (Little & Rubin, 2019). (2) Patterned responses: obvious fixed patterns such as “11111” or “12345” (Griffith & Peterson, 2006). (3) Short completion time: less than half the average time (M = 689.98 s, SD = 191.66 s), i.e., under 345 s, as such brevity strongly suggests random responding without careful reading. After screening, 1263 valid responses were retained, yielding a 95.68% valid response rate—well above the 70% reference threshold (Bai & Du, 2017)—thus providing a reliable basis for the study’s conclusions. Among the valid participants, 464 were male and 799 were female, aged 17–23 years (M = 18.56, SD = 0.904); 505 were from rural areas and 758 from urban areas; 1208 reported no Adverse childhood experiences (ACEs), while 235 (18.60%) had such experiences. Detailed demographic information is presented in Table 1.

### 2.2. Measures

#### 2.2.1. Adverse Childhood Experiences (ACEs)

Adverse childhood experiences were assessed using the Adverse Childhood Experiences Questionnaire adapted from Felitti et al. (1998). These indicators were assessed through self-report questionnaires, encompassing various childhood adversities, including supervisory neglect, emotional neglect, physical abuse, emotional abuse, sexual abuse, parental alcohol misuse, parental separation or divorce, household adult incarceration, and direct witnessing of violence. The Cronbach’s α for the questionnaire in our present sample was 0.816. In our study, ACEs is a binary variable: if an individual reported experiencing any of the adversities, they were considered exposed to ACEs (coded as 1). Conversely, if they reported no experiences of ACEs, they were coded as 0.

#### 2.2.2. Perceived Everyday Discrimination (PED)

Perceived everyday discrimination was assessed using the Perceived Everyday Discrimination Scale with six binary indicators, aligned with the items used by Nkwata et al. (2020). These items capture concerns and chronic stress related to perceived discrimination. Sample items include: “You receive poorer service than other people at restaurants or stores” and “You are treated with less courtesy or respect than other people.” Each indicator was coded as 0 (did not experience) or 1 (experienced), and the total score was summed to create a continuous variable ranging from 0 to 6, with higher scores indicating greater perceived everyday discrimination. To examine the construct validity of the Perceived Everyday Discrimination (PED) scale, we conducted an exploratory factor analysis. The results indicated that the Kaiser–Meyer–Olkin (KMO) measure of sampling adequacy was 0.721, indicating that the sample data is suitable for factor analysis. Bartlett’s test of sphericity yielded a chi-square value of 886.676, with 15 df and a significance level of *p* < 0.001 Additionally, the six items of the PED scale loaded onto a single component, and the component matrix shows factor loadings ranging from 0.521 to 0.695. The result provides support for the construct validity of the scale. The Cronbach’s α for the questionnaire in our present sample was 0.624.

#### 2.2.3. Shift-and-Persist Strategy

We measured the “shift-and-persist” strategy using the Shift-and-Persist Scale revised by E. Chen et al. (2015), Kallem et al. (2013), and Lam et al. (2018). This questionnaire comprises two dimensions: “shift” and “persist.” The “shift” dimension includes nine items, such as “When stressful events occur in my life, I consider what I can learn from them.” The “persist” dimension consists of seven items, including “I feel my life has a sense of purpose.” Participants rated their agreement with each item on a 4-point scale (ranging from “Completely Inconsistent” to “Completely Consistent”). We calculated the “shift-and-persist” score by averaging the separate scores for “shift” and “persist” and then summing them. A higher score indicates greater utilization of the strategy. The Cronbach’s α for the questionnaire in our present sample was 0.965.

#### 2.2.4. Depression Assessment

We assessed depression using the Chinese version of the Beck Depression Inventory-II (BDI-II-C), as revised by Z. Wang et al. (2011). This 21-item scale evaluates the severity of depressive symptoms over the past two weeks. Each item is scored on a scale of 0 to 3, resulting in a total score ranging from 0 to 63. The Cronbach’s α for the questionnaire in our present sample was 0.962.

### 2.3. Procedure

This study was approved by the Ethics committee of Wuhan University (IRB NO.WHU-HSS-2024002). Participants provided informed consent and were informed of their right to withdraw from the assessment at any time. They completed an anonymous self-report questionnaire, including demographic information and responses to all survey items. The entire process took approximately 20 min, and participants received compensation upon completion. We thoroughly checked all questionnaires for completeness.

### 2.4. Data Analysis

We conducted all analyses using SPSS 26. Prior to analysis, we standardized continuous variables to minimize multicollinearity and ensure comparability across different scales. Standardization helps to center the data around a mean of zero and a standard deviation of one, which can improve the stability of regression coefficients and enhance the interpretability of the results (Dormann et al., 2013; Kalnins, 2018). Our initial steps involved descriptive statistics and Pearson correlation analysis to explore the relationships among adverse childhood experiences (ACEs), perceived everyday discrimination (PED), the “shift-and-persist” coping strategy, and depression. To examine a moderated mediation model, we employed the SPSS PROCESS macro. Notably, previous research has established associations between age, gender, income, and depressive symptoms (Ohannessian et al., 2017; Ren et al., 2020; Salk et al., 2017; Whitaker et al., 2021). Furthermore, in our study, the results of the correlational analyses indicated that age and gender were significantly associated with depressive symptoms. Consequently, we included all participant characteristics including age, gender, income and household registration as covariates in our models and every path for control purposes.

We hypothesize that the “shift-and-persist” strategy may moderate the relationship between ACEs and PED, thereby influencing the link between ACEs and depression. To test these hypotheses, we followed Hayes’ (2017) method for examining moderated mediation. Given the non-normal distribution of scores on most ACEs scales (Tabachnick et al., 2013), we utilized the bias-corrected percentile bootstrap method for analysis (Kelley, 2005). Specifically: We first employed the SPSS PROCESS macro (Model 1) to assess whether the moderation effect of “shift-and-persist” on the relationship between adverse childhood experiences and depression is significant. Next, using the SPSS PROCESS macro (Model 8), we tested whether the coefficients of the mediation paths remain significant after adding the mediator variable. Additionally, we examined whether the interaction term significantly impacts the paths to PED and depression.

## 3. Results

### 3.1. Test for Common Method Bias

The potential for common method bias, due to the questionnaire-based data collection, was addressed through procedural controls. During the questionnaire administration, we emphasized anonymity, confidentiality, and the exclusive academic research purpose. Subsequently, we evaluated the effectiveness of these procedural controls using Harman’s single-factor test (Podsakoff et al., 2003). The analysis yielded nine factors with eigenvalues exceeding 1, and the first factor accounted for 27.71% of the variance, well below the 40% threshold, indicating no significant common method bias.

### 3.2. Descriptive Statistics

Table 2 presents the means, standard deviations, and inter-correlations among the variables under investigation. Notably, adverse childhood experiences exhibited a negative correlation with “shift-and-persist,” whereas it positively correlated with perceived everyday discrimination and depression. Simultaneously, the “shift-and-persist” construct demonstrated a negative correlation with perceived everyday discrimination and depression. Additionally, perceived everyday discrimination showed a positive correlation with depression.

### 3.3. Test for Moderation

We employed Model 1 from the SPSS PROCESS plugin to test moderation effects. The results show that ACEs significantly predicted depression (β = 0.30, t = 4.30, *p* < 0.001). The interaction between adverse childhood experiences and the “shift-and-persist” strategy significantly predicted depression (β = −0.24, t = −3.65, *p* < 0.001).

### 3.4. Test for Mediated Moderation

**Figure 2:**
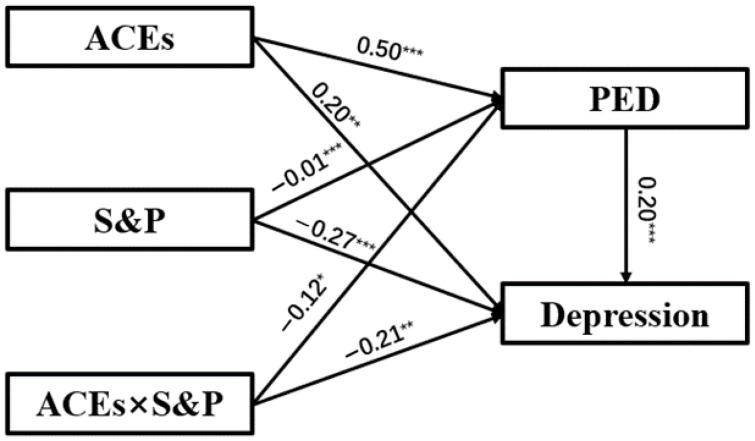
The mediated moderation model of the influence of adverse childhood experiences on depression. Note: * *p* < 0.05 ** *p* < 0.01 *** *p* < 0.001. ACEs = Adverse childhood experiences; PED = Perceived everyday discrimination; S&P = “shift-and-persist” strategy. Gender and age are control variables.

We employed Model 8 from the SPSS PROCESS plugin to test mediated moderation effects. The results supported our hypothesis (Table 3, Figure 2). Firstly, the interaction effect of ACEs and “shift-and-persist” strategy significantly predicted depression (β = −0.21, t = −3.21, *p* < 0.01) and PED (β = −0.16, t = −2.37, *p* < 0.05). This suggests that the “shift-and-persist” strategy moderates the relationship of adverse childhood experiences on depression and PED. Furthermore, ACEs positively predicted PED (β = 0.51, t = 6.93, *p* < 0.001) and depression (β = 0.20, t = 2.85, *p* < 0.01), and PED positively predicted depression (β = 0.20, t = 7.52, *p* < 0.001). This suggests that the mediating effect of PED is significant. Thus, the mediated moderation model was supported.

We divided “shift-and-persist” into high and low groups based on one standard deviation and examined the Bootstrap 95% CI of the indirect effect. The result shows that when “shift-and-persist” scores are low, the mediating effect of perceived everyday discrimination is significant (95% CI = [0.07, 0.21]), while when “shift-and-persist” scores are high, the mediating effect of perceived everyday discrimination is significant and weaker (95% CI = [0.001, 0.15]). This suggests that the “shift-and-persist” strategy negatively influences depression indirectly by influencing PED.

**Figure 3:**
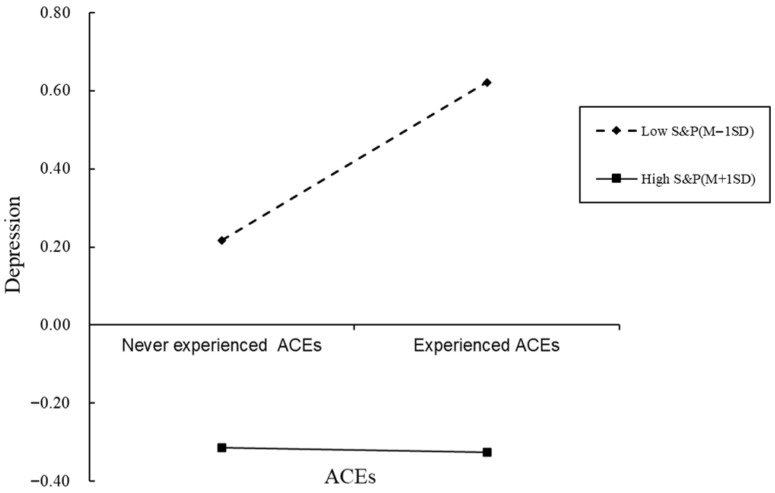
The “shift-and-persist” strategy as a moderator of the relationship between adverse childhood experiences and depression. Note: ACEs = Adverse childhood experiences; S&P = “shift-and-persist” strategy. Gender and age are control variables. The two regression lines represent the simple slopes at one standard deviation above the mean (high level, solid line) and one standard deviation below the mean (low level, dashed line) of the moderator.

For descriptive purposes, we conducted simple slope tests to examine the moderation effects of shift-and-persist strategy in the relationship between ACEs and depression (Figure 3). The result shows that for undergraduate students with low “shift-and-persist” strategy scores (1 SD below the mean), the association between ACEs and depression was significant (β = 0.40, t = 4.87, *p* < 0.001), whereas for undergraduate students with high “shift-and-persist” strategy scores (1 SD above the mean), the association between ACEs and depression was not significant (β = −0.01, t = −0.08, *p* > 0.05).

**Figure 4:**
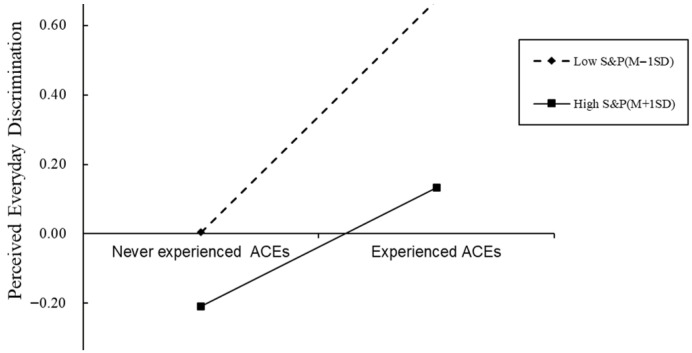
The “shift-and-persist” strategy as a moderator of the relationship between adverse childhood experiences and perceived everyday discrimination. Note: ACEs = Adverse childhood experiences; S&P = “shift-and-persist” strategy. Gender and age are control variables. The two regression lines represent the simple slopes at one standard deviation above the mean (high level, solid line) and one standard deviation below the mean (low level, dashed line) of the moderator.

For descriptive purposes, we conducted simple slope tests to examine the moderation effects of shift-and-persist strategy in the relationship between ACEs and PED (Figure 4). The result shows that for undergraduate students with low “shift-and-persist” strategy scores (1 SD below the mean), association between ACEs and PED was significant (β = 0.67, t = 7.68, *p* < 0.001), whereas for undergraduate students with high “shift-and-persist” strategy scores (1 SD above the mean), the association between ACEs and PED was much weaker (β = 0. 33, t = 2.98, *p* < 0.001).

## 4. Discussion

The link between ACEs and subsequent health challenges, particularly depression, is well-documented (Huh et al., 2017; Tachi et al., 2019; J. Wang et al., 2020; Windle et al., 2018). This study extends previous findings by examining the impact of ACEs on depression among Chinese college students and exploring the protective role of the “shift-and-persist” strategy. Consistent with prior research (Cavanaugh & Nelson, 2022; Desch et al., 2023; T. Zhang et al., 2023), our results indicate that ACEs are a significant predictor of depressive symptoms during university years. The advancement over prior research lies in the finding that the “shift-and-persist” strategy diminishes experiences of discrimination, which subsequently lowers depression levels in individuals with a history of ACEs.

### 4.1. The Mediating Effect of Perceived Everyday Discrimination

The mechanisms linking ACEs to depression remain to be fully elucidated, but there is evidence suggesting that ACEs predispose individuals to heightened stress sensitivity. Consequently, individuals may become more vigilant and sensitive to subsequent experiences of discrimination, expressing this sensitivity through an increased perception of themselves as targets of discrimination (Franklin & Boyd-Franklin, 2000; Matheson et al., 2019). Our main finding of a significant indirect effect, when statistically controlling for age, gender, household registration and income, points to the important role of perceived discrimination in the continuing influence of ACEs in adulthood across these groups.

This sensitivity is particularly relevant for college students, a population that experiences significant life changes, increased stress, and potential adverse experiences during their transition into adulthood. Evidence shows that depression often begins between adolescence and early adulthood (Thapar et al., 2012), and the proportion of college students facing psychological well-being issues, including stress, anxiety, and depression, has significantly increased in recent years (I. H. Chen et al., 2023; X. Liu et al., 2023; López Steinmetz et al., 2023).

While most previous studies on the health effects of discrimination have focused on “minority populations,” such as immigrants (Tsai et al., 2024), ethnic minorities (Christophe et al., 2019), and sexual minorities (Hollinsaid et al., 2023), as they are frequently exposed to discrimination. However, it is important to note that members of the so-called “majority group” can also experience discrimination (Lee & Khalid, 2016; Williams et al., 1997). Therefore, examining the impact of perceived everyday discrimination (PED) among general college students is crucial.

Additionally, ACEs may influence individuals’ responses in interpersonal relationships, intensifying sensitivity to rejection (Bombay et al., 2014). The lack of a supportive family environment in childhood may also impact individuals’ perception of themselves and their interpersonal relationships (Barrett & Fish, 2014). These research findings point to the predictive effect of ACEs on an individual’s perception of daily stressors, particularly those associated with interpersonal dynamics. Our study reveals that ACEs not only robustly predict depressive symptoms but also amplify the perception of PED later in life. Specifically, PED mediates the relationship between ACEs and depression. This suggests that an individual’s subjective perception of stress plays a crucial role in the link between ACEs and poor mental health (Kong et al., 2021; Nurius et al., 2016). 

Adverse childhood experiences (ACEs) may have widespread negative effects on mental and physical health throughout an individual’s lifespan (Campbell et al., 2016; Dube et al., 2003; Felitti et al., 1998; Hu et al., 2021). However, it is important to note that not all individuals exposed to ACEs will necessarily develop depression or experience poor health later in life. Protective factors exist that can mitigate the negative impact of ACEs. Previous research has identified several such protective factors, including psychological resilience (Zheng et al., 2022), positive childhood experiences (Qu et al., 2022), and positive parenting practices (Yamaoka & Bard, 2019).

Based on extensive empirical research, experts believe that the “shift-and-persist” strategy serves as both a down-regulation mechanism for stress-response adaptations and a protective factor against the impact of stress on health (E. Chen, 2012; E. Chen & Miller, 2012). In simpler terms, the “shift-and-persist” strategy influences an individual’s perception of stress, leading to reduced acute physiological activation of the hypothalamic–pituitary–adrenal (HPA) axis. Over time, this strategy helps prevent the development of pathogenic processes and ultimately reduces the risk of diseases triggered by uncontrollable stressors (Campbell et al., 2020; E. Chen & Miller, 2012; Ertekin et al., 2021). According to this theory, the “shift-and-persist” strategy can protect individuals’ health by minimizing their perception of stress, especially among those with ACEs.

However, despite its importance, previous research has rarely explored the cognitive mechanisms underlying the effectiveness of the “shift-and-persist” strategy. In our present study, we discovered that this strategy has a protective effect in the context of ACEs and depression. Specifically, when the “shift-and-persist” level is high, ACEs do not significantly predict depression. Conversely, when the “shift-and-persist” level is low, ACEs become a significant predictor of depression. Additionally, the “shift-and-persist” strategy operates by reducing the perceived level of daily discrimination among individuals with ACEs, thereby weakening the connection between ACEs and depression. These findings significantly expand the application domain of the “shift-and-persist” strategy.

### 4.2. Limitations and Future Directions

Previous research suggests that the “shift-and-persist” strategy does not correlate with improved physical or mental health in the absence of uncontrollable stressors (E. Chen, 2012; Christophe et al., 2019; Lam et al., 2018). Contrary to these findings, our study revealed that individuals employing a high level of the “shift-and-persist” strategy reported fewer depressive symptoms and lower psychological distress, even without exposure to adverse childhood experiences (ACEs). Furthermore, the “shift-and-persist” strategy appears to moderate the relationship between ACEs and psychological distress. Specifically, college students with low “shift-and-persist” strategy scores showed a significant effect of ACEs on psychological distress (β = 0.67, t = 7.68, *p* < 0.001). In contrast, for those with high “shift-and-persist” strategy scores (1 SD above the mean), the impact of ACEs was considerably weaker (β = 0.33, t = 2.98, *p* < 0.001), although still significant.

Regarding the inconsistent finding that the “shift-and-persist” strategy can protect individuals not exposed to ACEs, we propose that profound cultural influences may serve as a pivotal explanation. Existing empirical studies on this strategy predominantly rely on Western samples (Benner et al., 2024; E. Chen et al., 2023; López-Cepero et al., 2024), while its applicability in Asian contexts, particularly within Chinese culture, remains insufficiently validated. Consequently, the prevailing Western perspective that the strategy is effective solely for uncontrollable stressors cannot be directly extrapolated to the Chinese context. Chinese culture has long embraced values closely aligned with the “shift-and-persist” approach, exemplified by the adage “chī dé kǔ zhōng kǔ, fāng wéi rén shàng rén” (enduring hardship prepares one for future success), which embodies stress acceptance (“shift”) and goal persistence (“persist”) (Leung & Shek, 2013; Shek et al., 2003). Such cultural norms may elevate this strategy beyond a “crisis-coping tool” to become a generalized resource for daily stress management. Recent research on Chinese adolescents further supports this view: L. Chen et al. (2025) found that individuals with high levels of shift-and-persist exhibited significantly lower depression regardless of subjective socioeconomic status. Notably, higher socioeconomic groups typically encounter fewer uncontrollable stressors (E. Chen & Miller, 2012; Lam et al., 2018), suggesting that the strategy may confer broad-spectrum protective effects against diverse stress types within Chinese culture. These findings not only provide cultural and empirical evidence for the observed protective effects of shift-and-persist even in ACEs-free contexts but also highlight the need for future research to explore the interplay between cultural values and shift-and-persist strategies in shaping mental health protective mechanisms.

Individuals often face complex social environments laden with uncontrollable stressors such as poverty and economic pressures. Both positive and negative factors within one’s living environment can significantly influence health development (Bethell et al., 2019; Crouch et al., 2019). We found that the interaction term of ACEs and “shift-and-persist” strategy becomes more significant when the PED is added. This phenomenon can be understood as when a mediator related to perceived everyday discrimination or other stress-related factors is added, it may further highlight or reveal the moderating effect of the “shift-and-persist” strategy, leading to a stronger association between the moderator and the outcome. The concept of the “shift-and-persist” strategy was initially developed to address the detrimental effects of low socioeconomic status (SES) on health (E. Chen & Miller, 2012). Recent studies indicate that SES moderates the link between ACEs and health outcomes (Currie et al., 2021; Hanć et al., 2022; Sundel et al., 2018). Higher educational attainment has been shown to buffer the negative impacts of adverse childhood conditions on health later in life (Ding & He, 2021; Sheikh, 2018). Large-scale European surveys have also highlighted considerable differences in how multiple discriminations affect depression across countries. In regions with better socioeconomic conditions, the influence of multiple discriminations on depression is reduced (Alvarez-Galvez & Rojas-Garcia, 2019). Additionally, individuals with lower SES are more susceptible to everyday discrimination (Fuller-Rowell et al., 2018; Jokela & Fuller-Rowell, 2022) and are more likely to encounter ACEs (Straatmann et al., 2020). Thus, the increased significance of the moderation effect might reflect an indirect impact of the moderator on the outcome through the mediator under specific conditions, akin to the complex protective mechanism observed in the “shift-and-persist” strategy amidst multiple stressors. The interplay of various life stressors can lead to intricate outcomes, warranting further exploration into how the “shift-and-persist” strategy functions amidst the interactive effects of ACEs, SES, and other uncontrollable stressors.

Furthermore, individuals acquire the “shift-and-persist” strategy from role models during early childhood and develop it in subsequent environments (E. Chen et al., 2013; E. Chen & Miller, 2012; Lee et al., 2022). Therefore, positive interventions can also offer protective effects on health for individuals who did not acquire this strategy in early childhood (E. Chen & Miller, 2012). Previous research has already shown that the levels of both “shift” and “persist” strategies in individuals can be enhanced through interventions. For instance, online interventions targeting individuals’ cognitive reappraisal skills increase participants’ inclination for comprehensive cognitive reappraisal in daily life. The training group, compared to the control group, demonstrates higher cognitive reappraisal scores, increased happiness (life satisfaction, self-esteem, optimism, positive emotions), and fewer negative emotional reactions after the intervention (Ranney et al., 2017). Creating and showcasing documentaries illustrating exemplary behavior and offering hope courses significantly increase optimism and future orientation among lower-class individuals (Lybbert & Wydick, 2018). In addition, structural and systemic changes that address the root causes of discrimination are essential to promoting long-term mental health and well-being across populations. Reducing discrimination at the societal level can alleviate the need for individuals to rely heavily on coping strategies, thereby fostering healthier communities. Thus, future studies could develop comprehensive psychological intervention programs to enhance the “shift-and-persist” strategy and actively work towards diminishing its prevalence. These interventions could target individuals who have experienced ACEs, ultimately serving to protect the psychological well-being of disadvantaged individuals.

## 5. Conclusions

This study investigated the effects of adverse childhood experiences (ACEs) on depression in college students and assessed the “shift-and-persist” strategy’s protective mechanisms. The findings indicate that a robust “shift-and-persist” strategy can weaken the link between ACEs and depression. Specifically, high levels of this strategy diminish the predictive power of ACEs on depression, whereas low levels of the strategy allow ACEs to significantly predict depressive symptoms. Furthermore, the “shift-and-persist” strategy appears to lessen perceived everyday discrimination (PED) in students with ACEs, consequently mitigating their symptoms of depression.

## Figures and Tables

**Table 1:** Participant Characteristics (N = 1263).

Variables	N	%
**Age**		
17	103	8.2
18	570	45.1
19	410	32.5
20	151	12.0
21	24	1.9
22	4	0.3
23	1	0.1
**Gender**		
female	799	63.3
male	464	36.7
**Household registration**		
Agricultural household registration	505	40
Non-agricultural household registration	758	60
**Per Capita Disposable Income**		
<3000 yuan	331	26.2
3000 yuan to 5000 yuan	104	8.2
5000 yuan to 12,000 yuan	428	34.2
12,000 yuan to 20,000 yuan	67	5.3
20,000 yuan to 30,000 yuan	97	7.7
30,000 yuan to 55,000 yuan	144	11.4
>55,000 yuan	92	7.3
**ACEs**		
Never experienced ACEs	1028	81.4
Experienced ACEs	235	18.6

**Table 2:** Descriptive statistics and correlations among variables of interest (N = 1263).

	M	SD	1	2	3	4	5	6
Gender ^a^	–	–	–					
Age	18.56	0.90	0.02	–				
ACEs ^a^	–	–	−0.01	0.01	–			
Shift-and-persist	5.99	1.14	0.03	−0.04	−0.17 **	–		
PED	0.28	0.74	0.04	−0.02	0.23 **	−0.17 **	–	
Depression	6.86	9.89	0.07 **	0.08 **	0.20 **	−0.36 **	0.28 **	–

**Notes:** ** *p* < 0.01. ACEs = Adverse childhood experiences; PED = Perceived everyday discrimination. ^a^ = dummy variables, boy = 0, girl = 1, Never experienced ACEs = 0, Experienced ACEs = 1.

**Table 3:** Testing the mediated moderation effect in the relation between adverse childhood experiences and depression (N = 1263).

Variable	Model 1		Model 2	
	(Dependent Variable: PED)		(Dependent Variable: Depression)	
	*β*	*t*	*β*	*t*
ACEs	0.50	6.89 ***	0.20	2.88 **
S&P	−0.10	−3.39 ***	−0.27	−9.27 ***
ACEs × S&P	−0.17	−2.43 *	−0.21	−3.21 **
PED			0.20	7.52 ***
GENDER	0.10	−0.44	0.15	2.80 **
AGE	−0.01	1.73	0.07	2.52 *
HR	−0.001	−0.02	−0.001	−0.020
INCOME	0.000	−0.62	0.000	−0.620
*R* ^2^	0.28		0.45	
*F*	21.69 ***		53.15 ***	

**Notes:** * *p* < 0.05. ** *p* < 0.01. *** *p* < 0.001. ACEs = Adverse childhood experiences; PED = Perceived everyday discrimination; S&P = “shift-and-persist” strategy; HR = Household register. Gender and age are control variables.

## Data Availability

Data are not publicly available due to the sensitivity of the research topics and ethical constraints. To ensure honest responses, participants were assured during informed consent that their raw data would not be shared publicly. However, the data may be made available to qualified researchers upon reasonable request, provided participant confidentiality is strictly maintained.

## Abbreviations

The following abbreviations are used in this manuscript:

ACEs	Adverse Childhood Experiences
PED	Perceived Everyday Discrimination
SNS	Sensorineural System
HPA	hypothalamic–pituitary–adrenal
S&P	shift-and-persist
KMO	Kaiser–Meyer–Olkin
BDI-II-C	Beck Depression Inventory-II
HR	Household register
